# Supplementary material for: A Near-Infrared Fluorescent and Photoacoustic Probe for Visualizing Biothiols Dynamics in Tumor and Liver
Source: Molecules. 2023 Feb 27;28(5):2229. doi: 10.3390/molecules28052229 (PMC10005096; doi:10.3390/molecules28052229)
Supplement: Supplementary file 1 [file molecules-28-02229-s001.zip › molecules-2172071-supplementary.pdf]

A Near-Infrared Fluorescent and Photoacoustic Probe for Visualizing Biothiols Dynamics in Tumor and Liver

Weizhong Ding [1,2], ShanKun Yao [1,2], Yuncong Chen [1,2,3,*], Yanping Wu [1,2], Yaheng Li [1,2], Weijiang He [1,3,*] and Zijian Guo [1,2,3,*]

[1] State Key Laboratory of Coordination Chemistry, School of Chemistry and Chemical Engineering, Nanjing University, Nanjing 210023, China

[2] Chemistry and Biomedicine Innovation Center (ChemBIC), Nanjing University, Nanjing 210023, China

[3] Nanchuang (Jiangsu) Institute of Chemistry and Health, Jiangsu, Nanjing 210000, China

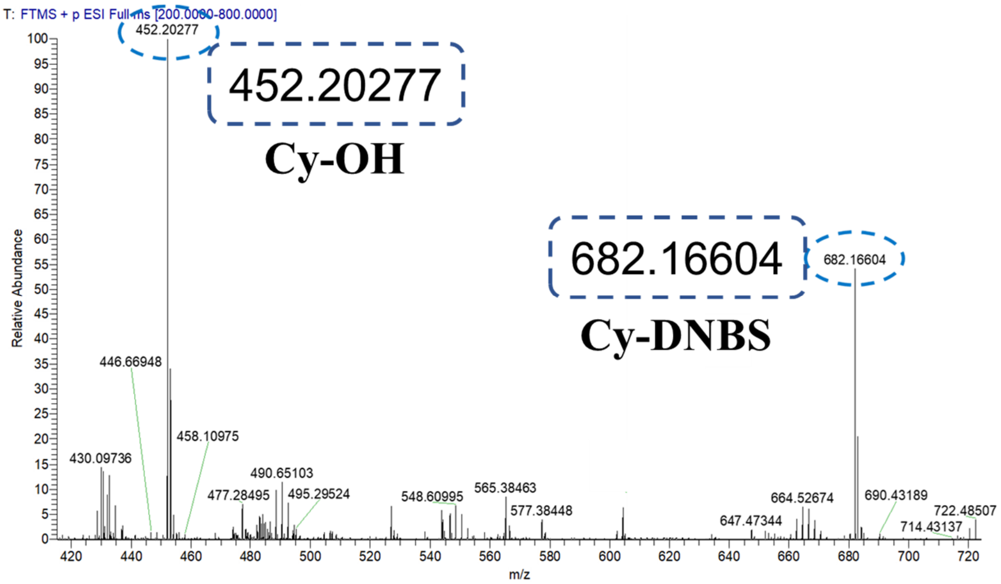

Figure S1. Mass spectrum acquired by mixed Cy-DNBS (10 µM) with GSH (20 µM) for 2 min and then determined by mass spectrometry with positive mode.

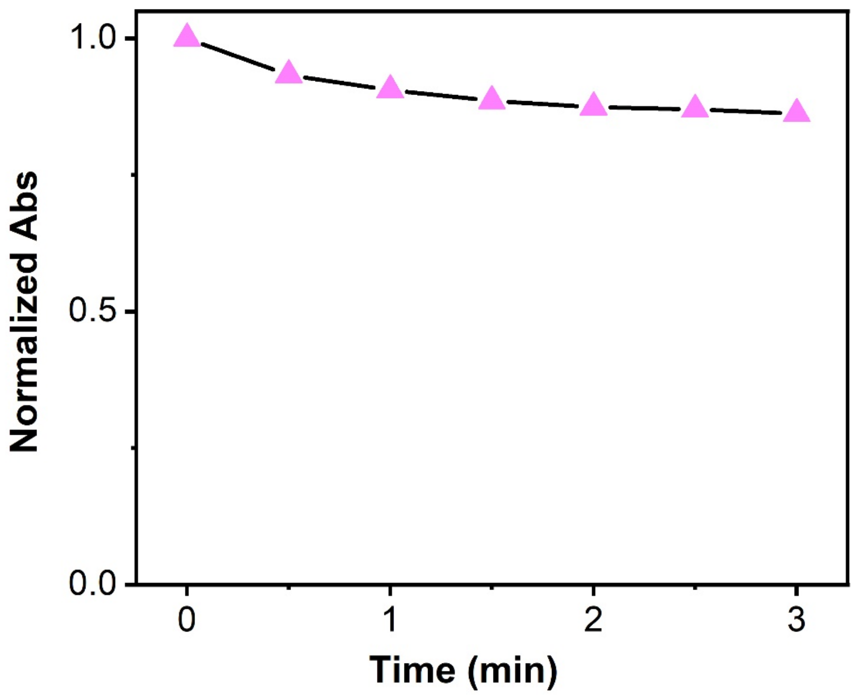

Figure S2. The photostability of Cy-DNBS (10 μM) in methanol was estimated by irradiating solutions in a quartz cuvette with a 635 nm laser (50 mW/cm[2]) for 3 min at a 30 second interval.

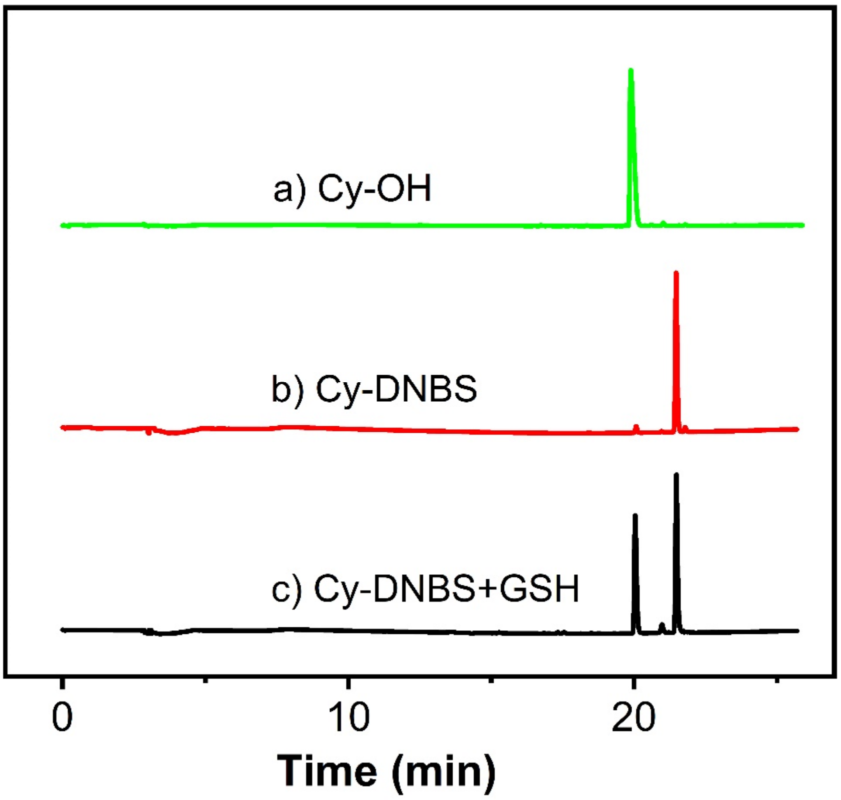

Figure S3. HPLC chromatograms of a) Cy-OH, b) Cy-DNBS and c) the mixtures of Cy-DNBS with GSH over 2 min. The mobile phase was acetonitrile and water.

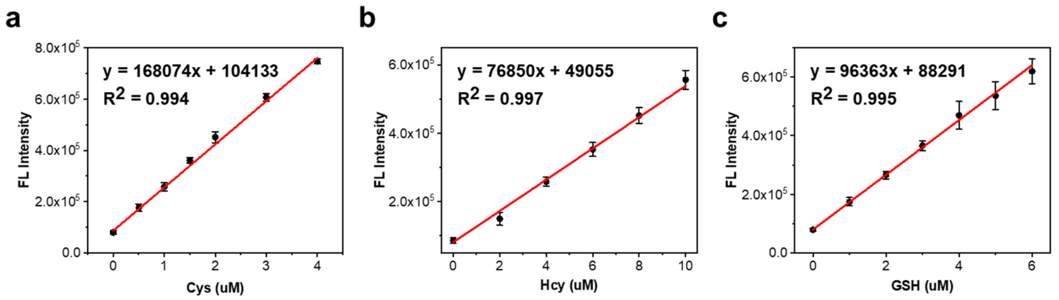

Figure S4. Linear relationship between fluorescence intensity of Cy-DNBS at 762 nm and concentration of (a) Cys, (b) Hcy and (c) GSH. λ_{ex} = 680 nm, λ_{em} = 700-900 nm. slit, 5 nm/5 nm.

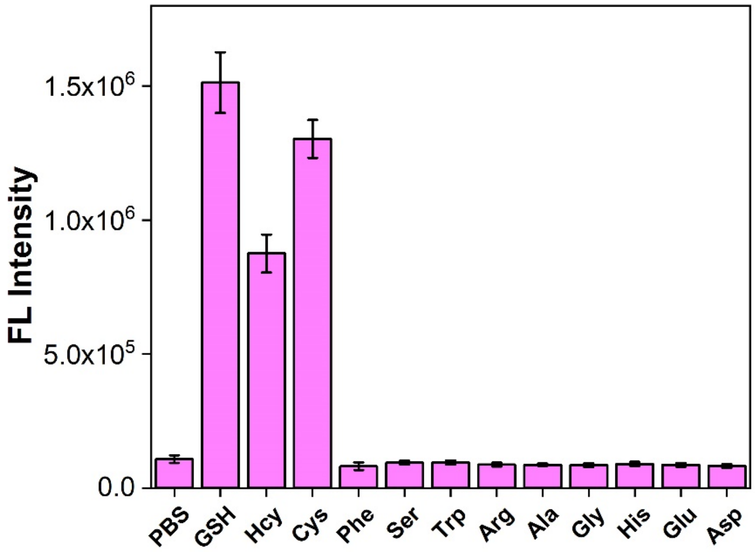

Figure S5. Histogram of the fluorescence intensity of Cy-DNBS (10 μM) towards Cys, Hcy, GSH and other amino acids including Phe, Ser, Trp, Glu, Gly, His, Ala, Arg, Asp (20 μM) at 762 nm in PBS solution (pH 7.40). λ_{ex} = 680 nm, λ_{em} = 700-900 nm. slit, 5 nm/5 nm.

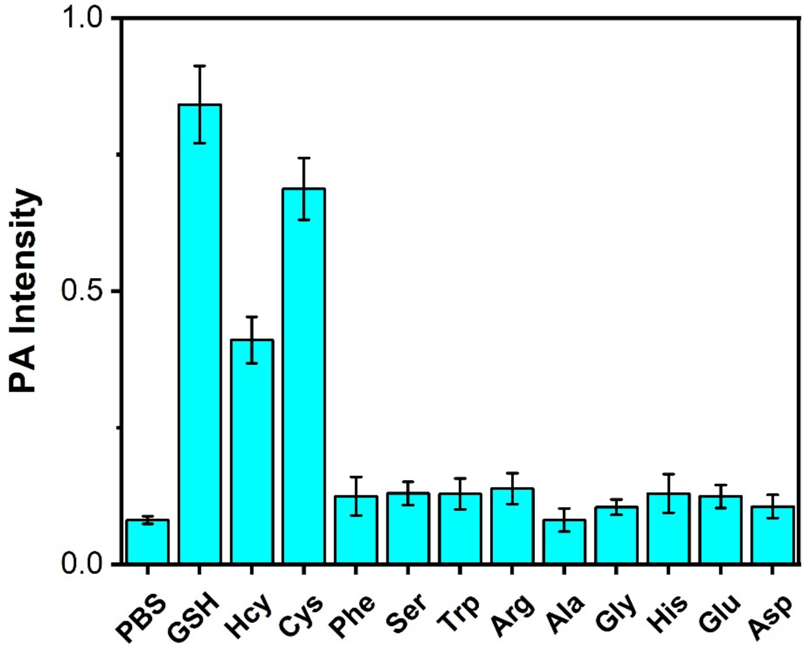

Figure S6. Histogram of the PA intensity of Cy-DNBS (10 μM) towards Cys, Hcy, GSH and other amino acids including Phe, Ser, Trp, Glu, Gly, His, Ala, Arg, Asp (20 μM) at 725 nm in PBS solution (pH 7.40).

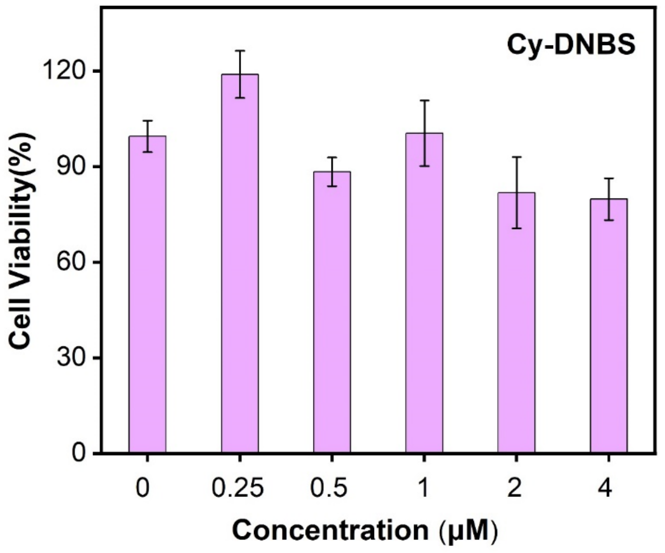

Figure S7. The cell viability of HepG2 cells after incubating with different concentration of Cy-DNBS were measured by MTT assay. The error bar represented the standard deviation of three individual measurements.

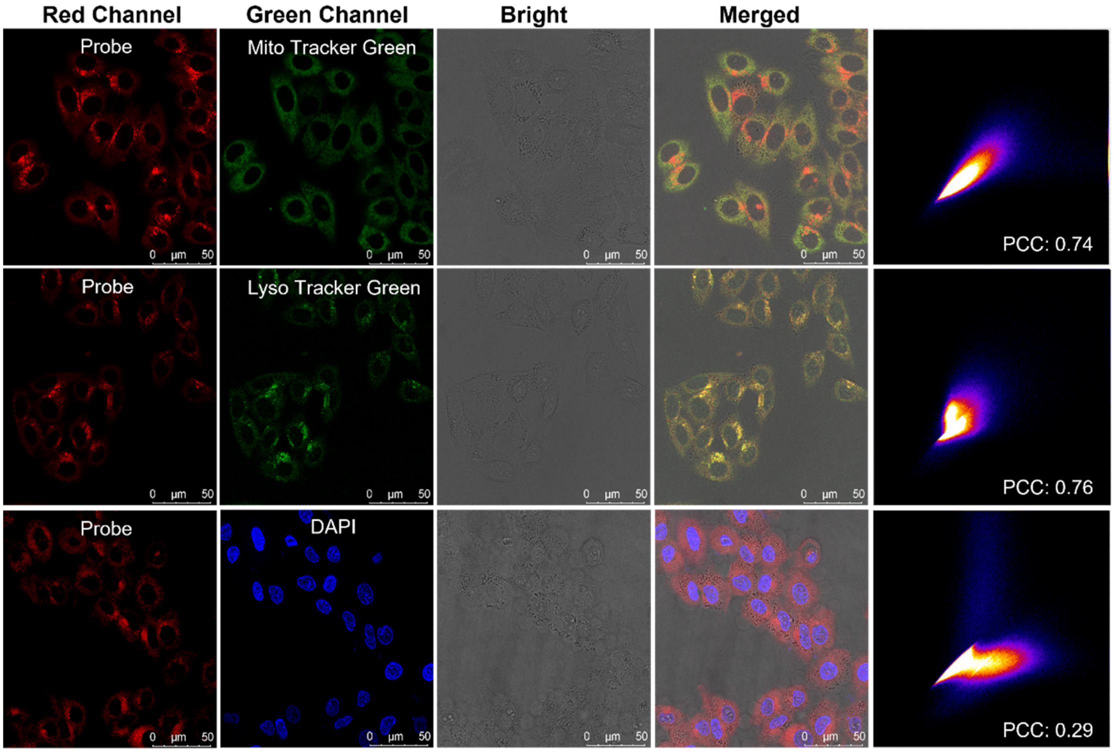

Figure S8. HepG2 cells were pretreated with exogenous Cys (2 mL, 4 μM) for 30 min and then Cy-DNBS (2 mL, 4 μM) were co-incubated with three commercial organelle dyes (Mito-Tracker, Lyso-Tracker and DAPI, 1 μM) for 30 min. Green channel (λ_{ex} = 488 nm, λ_{em} = 500-620 nm), Red channel (λ_{ex} = 633 nm, λ_{em} = 680-800 nm), Blue channel (λ_{ex} = 405 nm, λ_{em} = 420-550 nm). Scale bar: 50 μm.

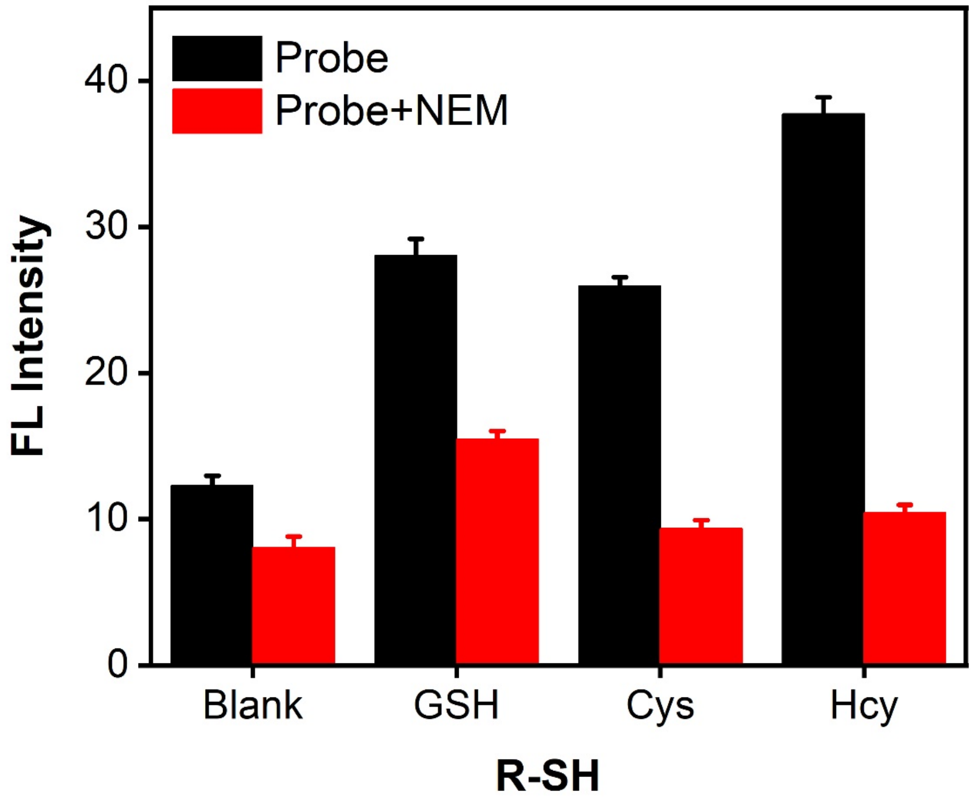

Figure S9. Fluorescence intensity data were calculated from **Figure 3**.

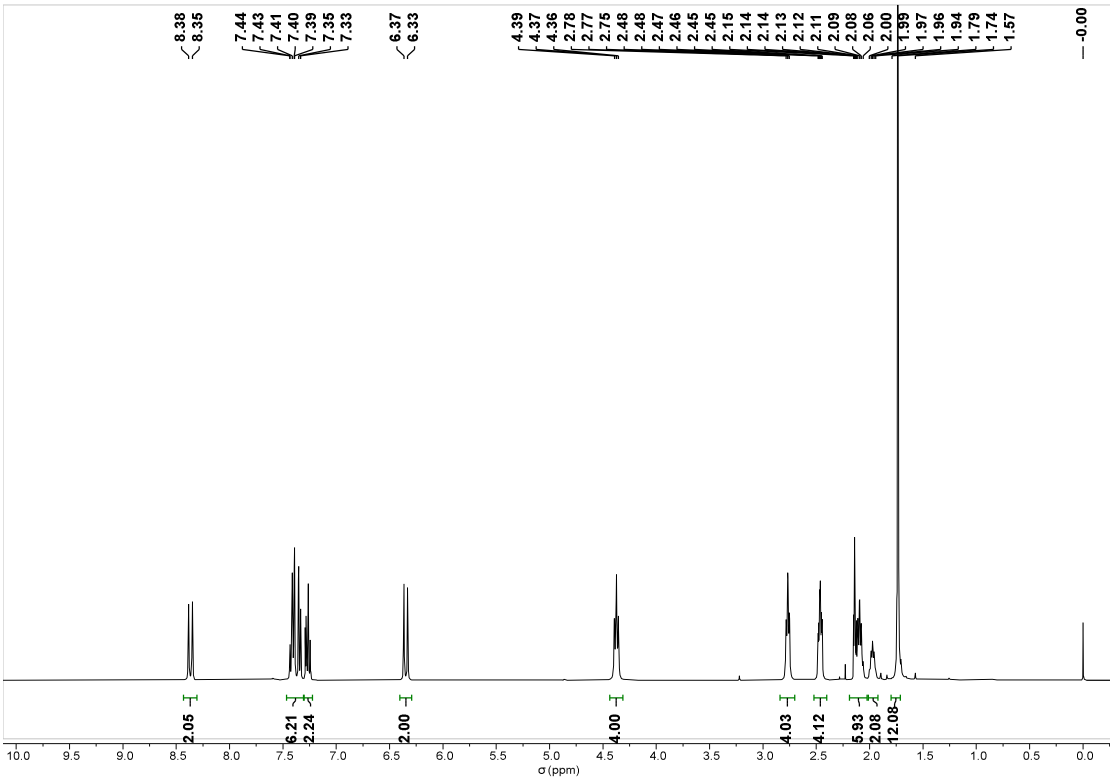

Figure S10. ^{1}H NMR spectrum of Cy7 in $CDCl_3$.

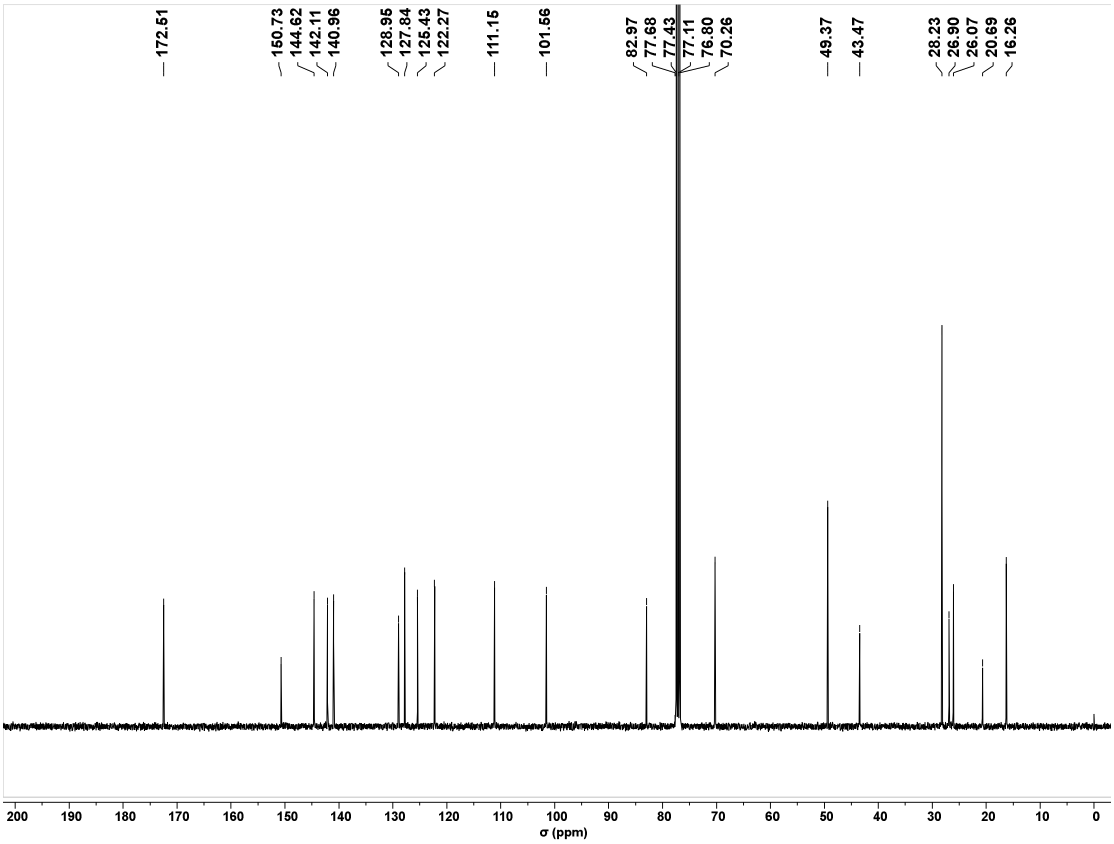

Figure S11. ^{13}C NMR spectrum of Cy7 in $CDCl_3$.

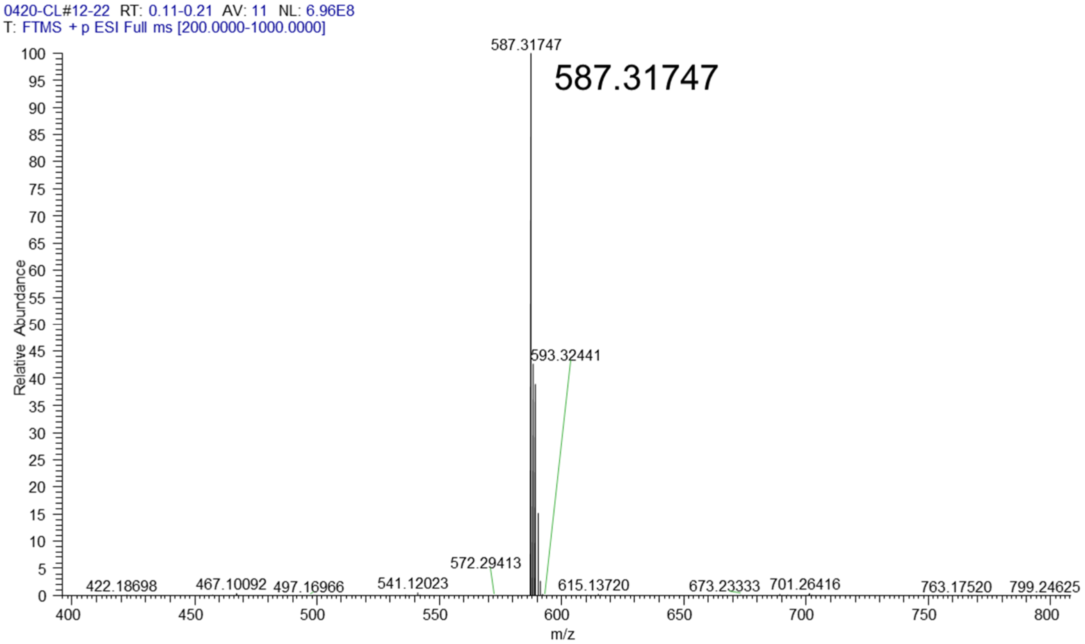

Figure S12. HR-MS spectrum of Cy7.

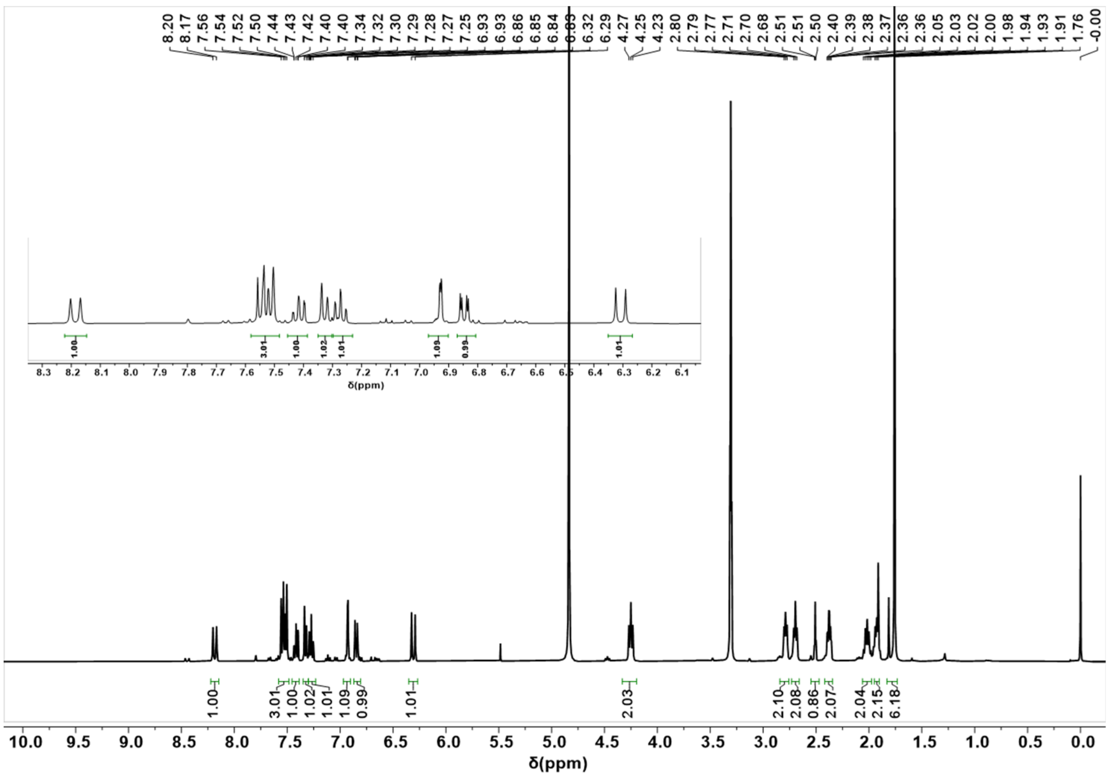

Figure S13. ^{1}H NMR spectrum of Cy-OH in CD_3OD.

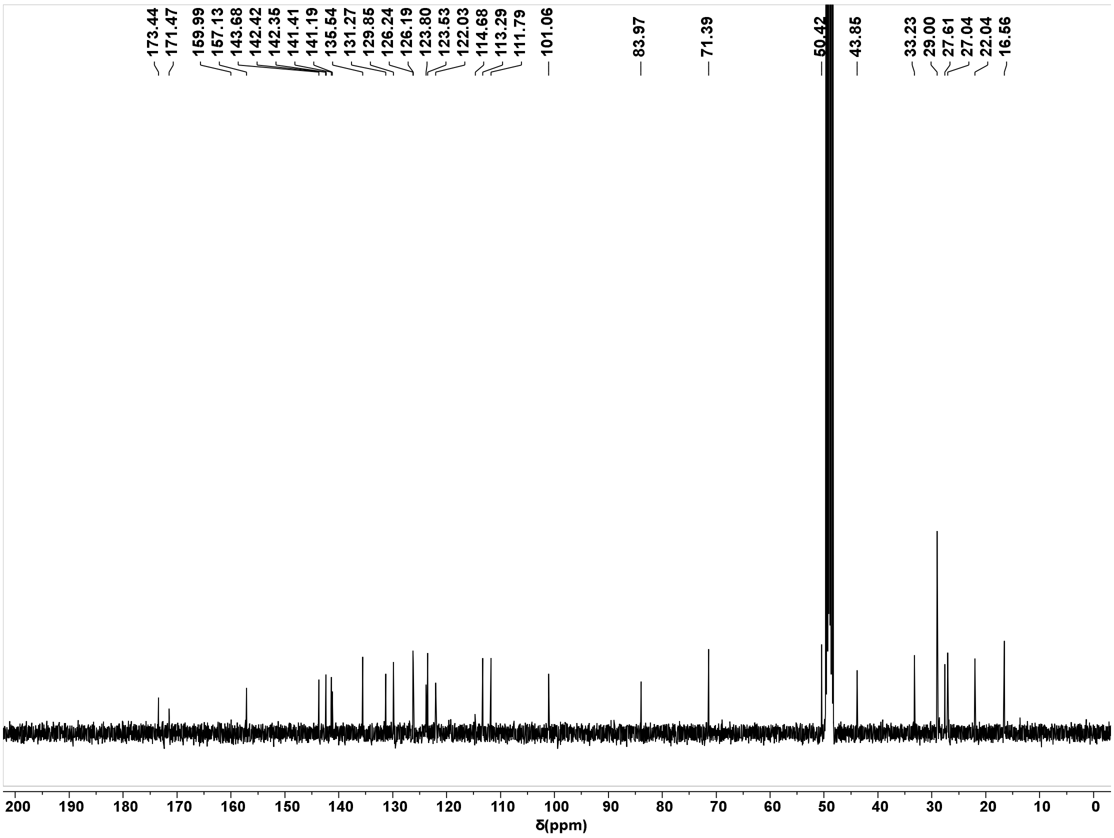

Figure S14. ^{13}C NMR spectrum of Cy-OH in CD_3OD.

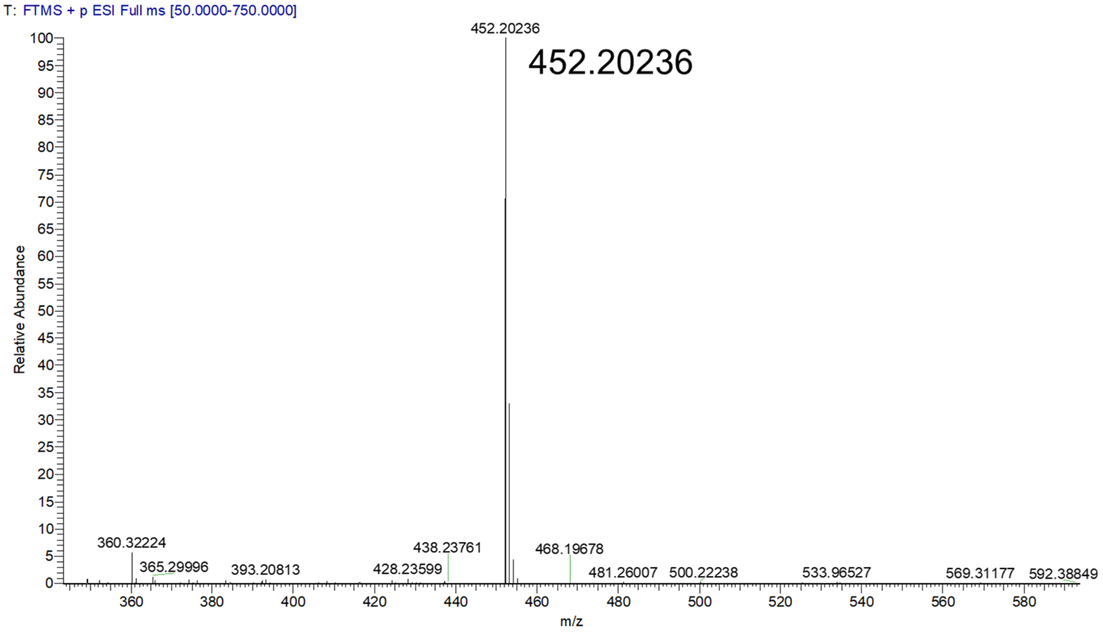

Figure S15. HR-MS spectrum of Cy-OH.

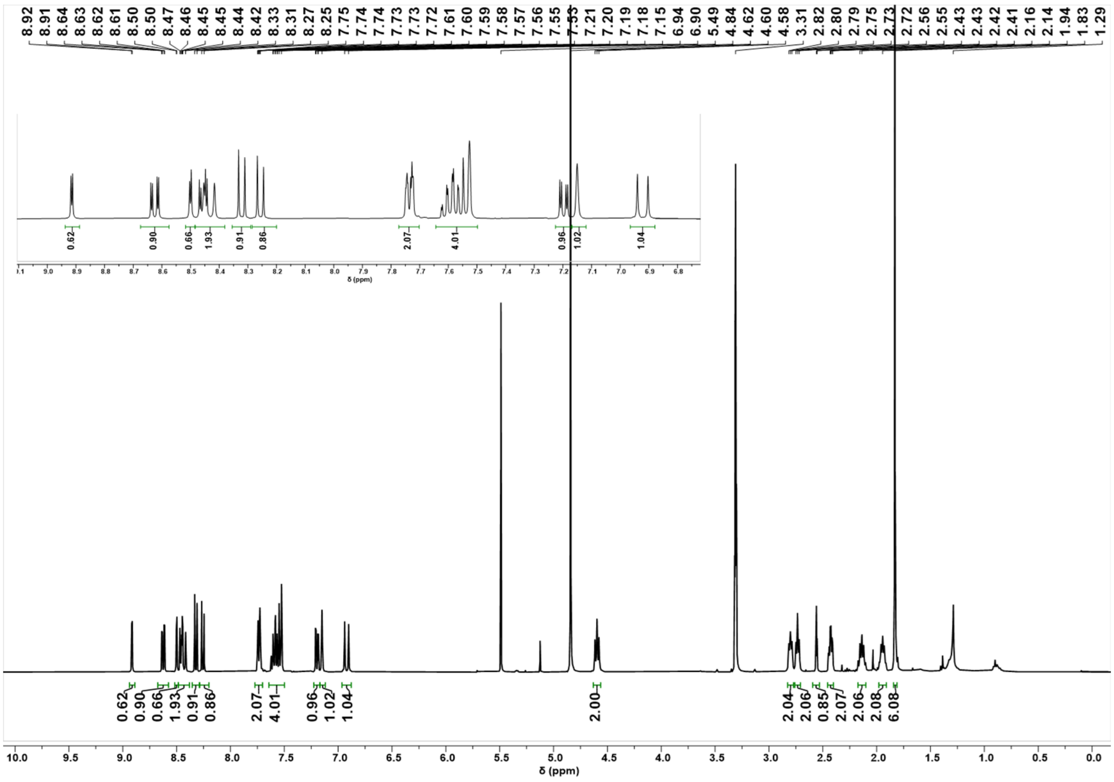

Figure S16. ^{1}H NMR spectrum of Cy-DNBS in CD_3OD.

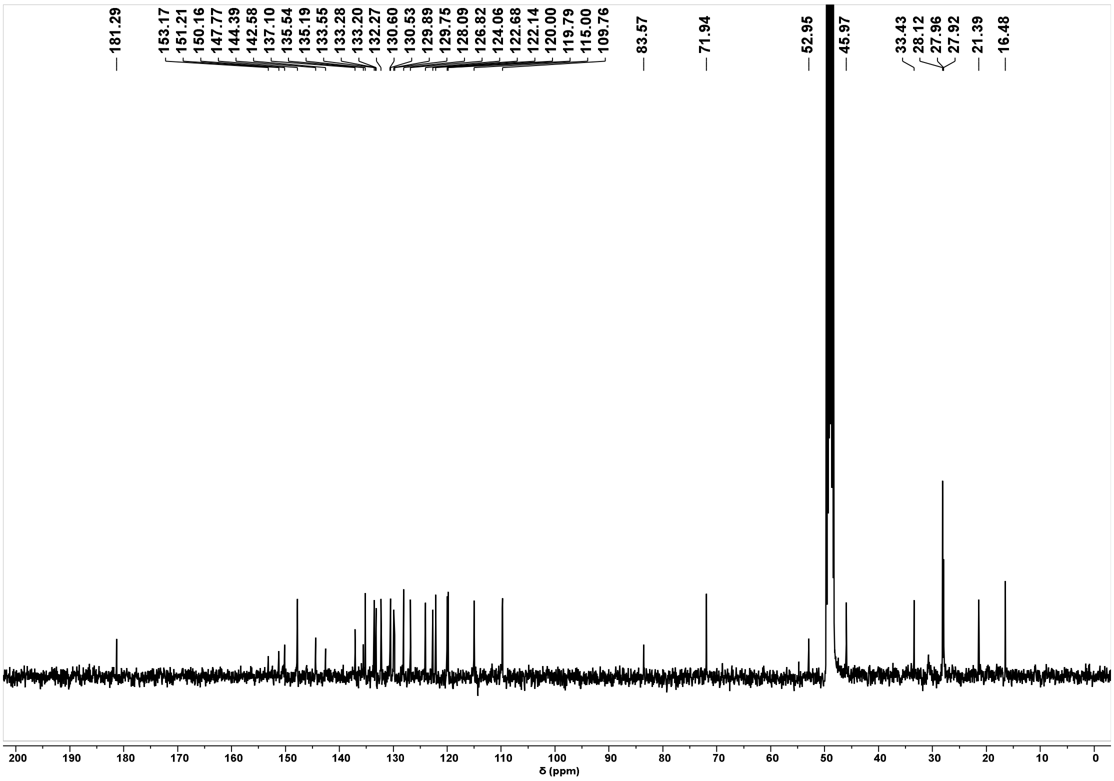

Figure S17. ^{13}C NMR spectrum of Cy-DNBS in CD_3OD.

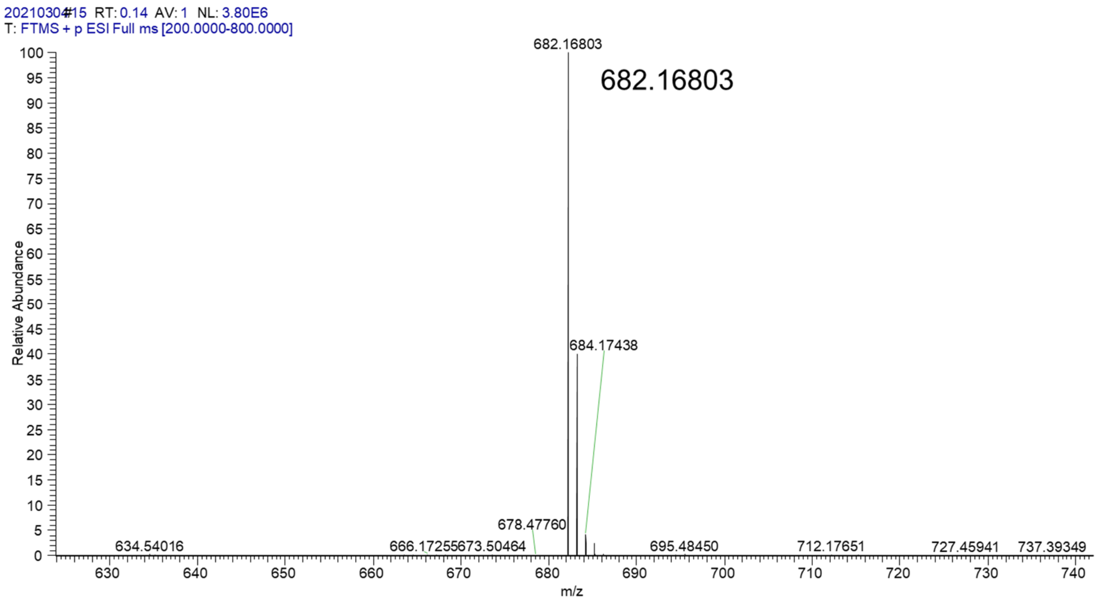

Figure S18. HR-MS spectrum of Cy-DNBS.

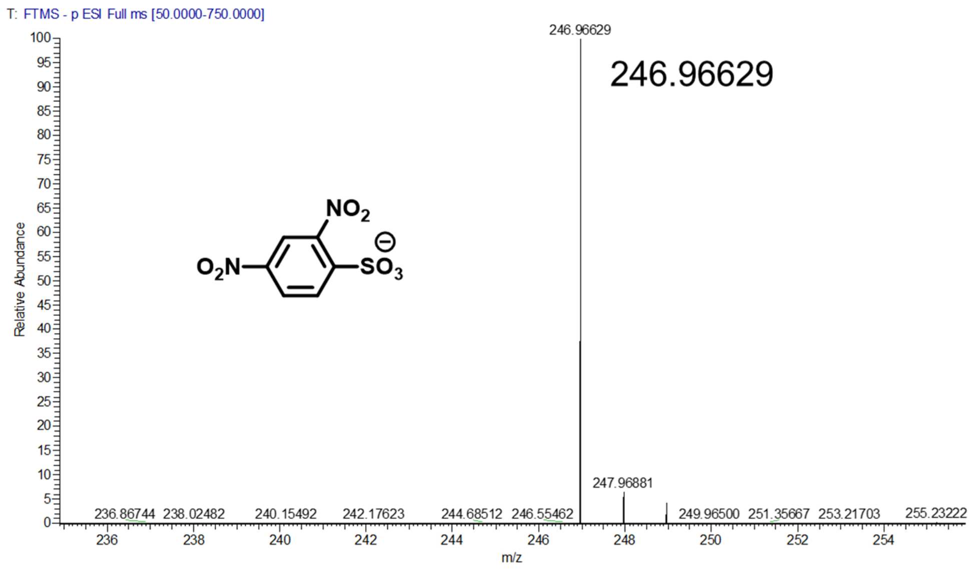

Figure S19. HR-MS spectrum of Cy-DNBS with negative mode.
